# Supplementary material for: Grammatical impairment in schizophrenia: An exploratory study of the pronominal and sentential domains
Source: PLoS One. 2023 Sep 12;18(9):e0291446. doi: 10.1371/journal.pone.0291446 (PMC10497169; doi:10.1371/journal.pone.0291446)
Supplement: S1 Appendix — (DOCX) [file pone.0291446.s001.docx]

**S1 Appendix: Annotation scheme and narrative examples of samples 1 and 2.**

**S1 Text 1:** **Annotation scheme.** Subject pronouns and sentences were manually annotated according to linguistic parameters adopted. Subject pronouns were divided into considering their phonological form (null (N) and overt (O)), person feature (first person (1P), second person (2P) and third person (3P))), referential status (referential (+R) and non-referential (-R)) and considering referential anomalies (anomalous (+A) and non-anomalous (–A)). Although the person feature of all subject pronouns was annotated, only null 3Person pronouns were statistically analyzed in relation to their referential status, and both null and overt 3Person referential pronouns were statistically analyzed in relation to referential anomaly. Sentences were classified in two main types: matrix (MS) and embedded (ES). Syntactically incomplete matrix and embedded sentences were further marked as truncated anomalous (TS+A) or non-anomalous (TS–A). For more details see Materials and Methods.

| **List of annotating marks** | |
| --- | --- |
| Overt Pronoun = O. | Matrix Sentence = [MS.] |
| Null Pronoun = N. | Embedded Sentence = [ES.] |
| First Person Pronoun = 1P. | Truncated Sentence = TS. |
| Second Person Pronoun = 2P. | Repetition of words etc. = {RP} |
| Third Person Pronoun = 3P. | Correction or complement of words etc. = {CR} |
| Referential Pronoun = +R. | Anomalous Truncated Sentences = +A |
| Non-referential Pronoun = -R. | Non-Anomalous Truncated Sentences = -A |
| Referentially Anomalous Pronouns = +A |  |
| Referentially Non-anomalous Pronouns = -A |  |

**S1 Text 2:** **narrative examples of samples 1 and 2.** We present examples of the original narratives – in Brazilian Portuguese –, literal English translations, and comments to the annotation marks. In the original narratives, null pronouns were represented as *pro*, and their equivalent subject pronouns were inserted in brackets in the translations. Missing interviewer’s comments were represented as […].

**Narrative examples of sample 1**

*Dream narrative of a NSZ participant:*

Interviewer: [...]

Participant: [^MS^ eu^O.1P.+R.-A^ tenho um sonho não tão recente] [^MS^ mas *pro*^N.3P.+R.-A^ é um sonho [^ES^ que eu^O.1P.+R.-A^ não esqueço]] [^MS^ os outros sonhos {^RP^ os outros sonhos} eu^O.1P.+R.-A^ já esqueci [^ES^ porque *pro*^N.3P.+R.-A^ têm passado]] ^[MS^ mas esses é um sonho [^ES^ que eu^O.1P.+R.-A^ não esqueço]] [^MS^ que *pro*^N.3P.-R^ é [^ES^ quando eu^O.1P.+R.-A^ sonho com o meu avô] [^MS^ que ele^O.3P.+R.-A^ já morreu]

Interviewer: [...]

Participant: [^MS^ ah digamos [^ES^ que [^MS^ deixa [^ES^ eu ver]] *pro*^N.3P.-R^ faz um {^RP^ um} mês {^RP^ um mês}]

Interviewer: [...]

Participant: é

Interviewer: [...]

Participant: [^MS^ todas as vezes [^ES^ que eu^O.1P.+R.-A^ sonho com ele] eu^O.1P.+R.-A^ não esqueço do sonho] [^MS^ *pro*^N.2P.+R.-A^ entendeu] [^MS^ os outros sonhos eu^O.1P.+R.-A^ até lembro alguns dias ainda] [^MS^ mas depois *pro*^N.1P.+R.-A^ acabo [^ES^ esquecendo]] [^MS^ mas o dele eu^O.1P.+R.-A^ não esqueço] [^MS.TS.+A^ eu^O.1P.+R.-A^ tenho uma ligação muito grande] [^MS^ eu^O.1P.+R.-A^ tinha uma ligação muito grande com ele] [^MS^ *pro*^N.1P.+R.-A^ considerava ele mais que meu pai] [^MS^ o amor o carinho por ele era muito grande]

Interviewer: [...]

Participant: [^MS^ *pro*^N.3P.-R^ fazem dois anos] [^MS^ *pro*^N.1P.+R.-A^ posso contar o sonho?]

Interviewer: [...]

Participant: [^MS^ bem eu^O.1P.+R.-A^ sonhei [^ES^ que ele^O.3P.+R.-A^ estava em um jardim] [^MS^ *pro*^N.1P.+R.-A^ não sei [^ES^ se você^O.2P.+R.-A^ lembra a lateral do prédio de Macaíba {^CR^ um jardim bem verde bem bonito ali}] pronto [^MS^ a minha impressão é [^ES^ que eu^O.1P.+R.-A^ estava ali em um jardim como aquele ali ] [^MS^ e de repente eu^O.1P.+R.-A^ via [^ES^ meu avô caminhando]] [^MS^ e ele^O.3P.+R.-A^ era deficiente meu avô] [^MS^ só que no sonho ele^O.3P.+R.-A^ não era deficiente] [^MS^ *pro*^N.3P.+R.-A^ era uma pessoa normal] [^MS^ deficiente ele^O.3P.+R.-A^ era deficiente de uma perna] [^MS^ *pro*^N.3P.+R.-A^ tinha uma perna amputada] [^MS^ aí eu^O.1P.+R.-A^ chegava] e [^MS^ *pro*^N.1P.+R.-A^ dizia] [^MS^ vovô o que é [^ES^ que o senhor está fazendo aqui]] [^MS^ aí ele^O.3P.+R.-A^ começava [^ES^ a rir]] e [^MS^ *pro*^N.3P.+R.-A^ dizia] [^MS^ não *pro*^N.1P.+R.-A^ vim só [^ES^ conversar um pouco com você]] [^MS^ aí no sonho eu^O.1P.+R.-A^ falava] [^MS^ vô mas eu^O.1P.+R.-A^ estou tão triste com alguma coisa [^ES^ que aconteceu]] [^MS^ eu^O.1P.+R.-A^ contava para ele uma coisa [^ES^ que tinha acontecido comigo]] [^MS^ aí ele^O.3P.+R.-A^ dizia assim] [^MS^ não fique triste [^ES^ porque eu^O.1P.+R.-A^ não gosto [^ES^ de ver [^ES^ você chorando]]]] [^MS^ *pro*^N.1P.+R.-A^ não gosto [^ES^ de ver você triste]] [^MS^ eu^O.1P.+R.-A^ sempre disse a você [^ES^ que a gente^O.1P.-R+GEN^ na vida tem [^ES^ que lutar {^CR^ lutar muito por tudo} [^ES^ que a gente^O.1P.-R+GEN^ quer]]]] [^MS^ aí eu^O.1P.+R.-A^ falava] [^MS^ *pro*^N.3P.-R^ está bom vô] [^MS^ mas eu^O.1P.+R.-A^ vou pedir uma coisa] [^MS^ não vá embora] [^MS^ aí ele^O.3P.+R.-A^ me abraçava] [^MS^ eu^O.1P.+R.-A^ segurava ele bem firme] e [^MS^ *pro*^N.1P.+R.-A^ dizia assim] [^MS^ vovô não vá {^RP^ não vá} embora] [^MS^ não me deixe] [^MS^ fique comigo] [^MS^ eu^O.1P.+R.-A^ preciso demais de você] [^MS^ a não ser [^ES^ que o senhor queira [^ES^ me levar]]] [^MS^ [^ES^ se o senhor quiser [^ES^ me levar]] eu^O.1P.+R.-A^ vou também] [^MS^ eu^O.1P.+R.-A^ prefiro [^ES^ ir do que ficar]] [^MS^ aí ele^O.3P.+R.-A^ dizia assim] [^MS^ mas você^O.2P.+R.-A^ tem uma criança] [^MS.TS.+A^ você^O.2P.+R.-A^ precisa] [^MS^ essa criança precisa de você] [^MS^ então você^O.2P.+R.-A^ não pode ir] [^MS^ aí ele^O.3P.+R.-A^ começava [^ES^ a caminhar]] e [^MS^ eu^O.1P.+R.-A^ via bem o Antônio {^CR^ o Antônio do trabalho}] [^MS^ ele^O.3P.+R.-A^ dizia] [^MS^ *pro*^N.3P.+R.-A^ é seu avô?] [^MS.TS.-A^ eu^O.1P.+R.-A^ dizia] [^MS^ mas eu^O.1P.+R.-A^ achava [^ES^ que só eu^O.1P.+R.-A^ que estava vendo ele]] [^MS^ mas o Antônio também via] [^MS^ então ele^O.3P.+R.+A^ desaparecia] e [^MS^ eu^O.1P.+R.-A^ acordava]

Translation: - […] - I have a dream not so recent, but (it)^1^ is a dream that I don’t forget. The other dreams… the other dreams^2^, I already forgot, because (they)^1^ have a past. But these is a dream that I don’t forget. (It)^3^ is when I dream with my grandpa that he has already died. - […] - ah, lets’ say that… let me see... (it)^3^ has been a… a^2^ month… a month^2^. - […] - (it) is^4^. - […] - Every time that I dream with him, I don’t forget the dream. Do (you) understand? The other dreams, I remember for a few days. But then (I) end up forgetting. But the one with him I don’t forget. I have a very strong relation (?)^5^. I had a very strong relation with him. (I) considered him more than my father. The love, the caring for him was very strong. - […] - (It)^3^ has been two years. Can (I) tell the dream? - […] - Well, I dreamt that he was in a garden. (I) don’t know if you remember the side of the Macaíba building… a garden very green^6^, very beautiful there. Right. My impression is that I was there in a garden like that one. And suddenly I saw my grandpa walking. And he was disabled my grandpa. Only that in the dream he wasn’t disabled. (He)^1^ was a normal person. Disabled, he was disabled of one leg. (He)^1^ had one leg amputated. Then I arrived, and (I) said: grandpa, what is it that you are doing here? Then he started to laugh, and (he)^1^ said: no, (I) came just to talk a little with you. Then, in the dream, I said: grandpa, but I am so sad with something that happened. I told him something that had happened to me. Then he said this: I don’t like to see you crying. (I) don’t like to see you sad. I always told you that, in life, we have to fight… fight a lot^6^ for everything that we want. Then I said: (it)^3^ is alright grandpa. But I will ask something. Don’t go away. Then, he hugged me. I held him very tight, and (I) said this: grandpa, don’t go, don’t go^2^ away. Don’t leave me. Stay with me. I need you too much. Unless you want to take me. If you want to take me, I will go too. I’d rather go than stay. Then, he said this: but you have a child. You must (?)^5^… This child needs you. So, you cannot go. Then, he started to walk. And I saw, right there, Antônio… Antônio from work^6^. And he said: is (he)^1^ your grandpa? I said (?)^5^. But I thought that it was only I that was seeing him. But Antônio also saw. Then, he^7^ disappeared. And I woke up.

Comments: ^1^null 3Person referentially non-anomalous pronouns; ^2^phrase/word repetition; ^3^null 3Person non-referential (expletive) pronouns; ^4^affirmative answer, which was not annotated; ^5^truncated anomalous sentences; ^6^correction or adding of information; ^7^overt 3Person referentially anomalous pronoun, its reference is ambiguous.

*Dream narrative of a patient with SZ:*

Interviewer: Então, me conta como é que foi esse sonho?

Participant: [^MS^ eu^O.1P.+R.-A^ {^RP^ eu^O.1P.+R.-A^} entrava numa igreja] [^MS^ uma mulher muito bonita arrumou um casamento] [^MS^ *pro*^N.3P.+R.-A^ ia ser muito feliz]

Interviewer: Que legal! Aí o que mais?

Participant: [^MS^ *pro*^N.3P.-R^ tem uma casa]

Interviewer: Tem uma casa, já. Que bom! A casa é como?

Participant: com internet de frente uma cisterna d’água

Interviewer: Eita! E essa casa era no sonho?

Participant: [^MS^ *pro*^N.3P.-R^ é só um sonho [^ES^ que eu^O.1P.+R.-A^ tenho]]

Interviewer: Só um sonho? Muito bem. Aí no sonho, vocês entravam na igreja?

Participant: era

Translation: - So, tell me how was that dream? - I… I^1^ walked into a church. A very pretty woman … arranged a wedding. (She)^2^ was going to be very happy. - That’s cool! What else? - (There)^3^ is a house. - There is a house already. How nice! How is the house? - with internet facing a water tank - Wow! And was this house in the dream? - (It)^3^ is just a dream that I have. - Hum. - (It)^3^ is just a dream. - Just a dream? Very good. And in the dream, did you enter the church? - (it) was^4^.

Comments: ^1^word repetition; ^2^null 3Person referentially non-anomalous pronoun, ^3^null 3Person non-referential (expletive) pronoun; ^4^affirmative answer, which was not annotated.

*Waking narrative of a NSZ participant:*

Interviewer: Não. O dia antes de ter tido o sonho.

Participant: um dia antes sim normal [^MS^ *pro*^N.1P.+R.-A^ conversei com minha família {^CR^ com mamãe tudinho} [^MS^ *pro*^N.1P.+R.-A^ fui dormir] [^MS^ *pro*^N.1P.+R.-A^ dei boa noite pra minha mãe tudo ] [^MS^ e ele^O.3P.+R.+A^ saiu] [^MS.TS.+A^ aí tipo assim eu^O.1P.+R.-A^ venho naquela] [^MS^ sonhando] e [^MS^ vendo [^ES^ aquelas coisa acontecendo] né [^MS^ *pro*^N.1P.+R.-A^ acordei assombrada] né

Translation: - No. The day before you had the dream. - One day before. Yes. Normal. I talked with my family, with mom and everyone^1^. (I) went to bed. I gave good night to my mom, all. And he^2^ left. Then, kinda, I come in that (?)^3^… Dreaming and seeing those things happening, alright. (I) woke up haunted, alright”.

Comments: ^1^correction or additional information; ^2^overt 3Person pronoun missing reference; ^3^truncated anomalous sentence.

*Waking narrative of a patient with SZ:*

Interviewer: […]

Participant: não [^MS^ eu^O.1P.+R.-A^ trabalhei ] [^MS^ *pro*^N.1P.+R.-A^ trabalhei o dia depois ] [^MS^ aí *pro*^N.3P.+R.+A^ teve muita dor na coluna [uma dor na cabeça]] [^MS^ como *pro*^N.3P.+R.+A^ está agora]] [^MS^ *pro*^N.3P.-R^ faz é tempo [^ES.TS.+A^ que *pro*^N.1P.+R.-A^ estou com uma]] [^MS^ *pro*^N.3P.+R.+A^ está doendo direto doutora] [^MS^ *pro*^N.1P.+R.-A^ fui me deitar] [^MS^ *pro*^N.1P.+R.-A^ tomei um comprimido] [^MS^ aí *pro*^N.1P.+R.-A^ estava dormindo] e [^MS^ *pro*^N.1P.+R.-A^ estava acordado]

Interviewer: […]

Participant: não só com o advogado mesmo

Translation: - [...] - No, I worked. (I) worked the day after. Then, (?)^1^ was much pain in the back, a pain in the head. Like (?)^1^ is now. (It) has been a long time that (I) am with a (?)^2^… (?)^1^ is hurting unstoppably doctor. (I) went to bed. I took a pill. Then, (I) was sleeping and (I) was awake. - [...] - No, only with the lawyer indeed.

Comments: ^1^null 3Person anomalous pronouns, its reference is missing; ^2^truncated anomalous sentence.

**Narrative examples of sample 2**

*Dream narrative of a patient with SZ:*

Interviewer: Certo, agora me conta o último sonho que você teve, mais recente?

Participant: [^MS.TS.+A^ Conversar com Deus] e [^MS.TS.+A^ fazer muita coisa]]. [^MS.TS.+A^ Rezar], [^MS.TS.+A^ ler bíblia], [^MS.TS.+A^ brincar], e [^MS.TS.+A^ fazer bastante coisa]. E muita coisa ainda [^ES.TS.+A^ que eu^O.1P.+R.-A^ fazia], [^MS.TS.+A^ fazer], [^MS.TS.+A^ conversar com muita coisa], [^MS.TS.+A^ conversar com cobras], e só.

Translation: - Right, now tell the last dream you had. The most recent one. - To talk^1^ to God and to do^1^ many thing. To pray^1^, to read^1^ the Bible, to play^1^ and to do^1^ a lot of thing. And many things still^2^ that I did, to do^1^, to talk^1^ to many things, to talk^1^ to snakes, and that’s all.

Comments: ^1^truncated anomalous sentence, ungrammatical use of the infinite verbal form; ^2^truncated anomalous sentence, unclear meaning.

*Dream narrative of a NSZ participant:*

Interviewer: Agora, conta pra mim o sonho mais recente que você lembra.

Participant: [^MS^ *pro*^N.1P.+R.-A^ acho [^ES^ que *pro*^N.3P.+R.-A^ foi semana passada] [^MS^ *pro*^N.3P.+R.-A^ era um sonho [^ES^ que era [^ES^ se eu estivesse em 2 corpos ao mesmo tempo]]] [^MS^ tipo eu^O.1P.+R.-A^ era um menino e uma menina ao mesmo tempo] e [^MS^ eles^O.3P.+R.-A^ estavam tentando [^ES^ se encontrar]] [^MS^ e *pro*^N.3P.-R^ era um lugar muito estranho tipo um labirinto] [^MS^ e eu^O.1P.+R.-A^ não conseguia [^ES.TS.+A^ encontrar]] [^MS^ e às vezes eu^O.1P.^ estava no corpo de uma pessoa e outras no de outra] [^MS^ e o sonho todo era [^ES^ tentando [^ES^ achar essas 2 pessoas]]] [^MS^ que eu^O.1P.+R.-A^ era ambas as pessoas]

Translation: - Right, now you tell me the most recent dream that you remember. - (I) think that (it)^1^ was last week. (It)^1^ was a dream that was as if I was in 2 bodies at the same time. Like, I was a boy and a girl at the same time, and they were trying to meet. And (it)^1^ was a very strange place, kind of a maze, and I couldn’t find (?)^2^. And, sometimes, I was in the body of one person, and, other times, of another. And the entire dream was trying to find these two people, that I was both people.

Comments: ^1^null 3Person non-referential (expletive) pronouns; ^2^truncated anomalous sentence, unclear meaning.

*Waking narrative of a patient with SZ:*

Interviewer: Então me conta como foi teu dia ontem.

Participant: [^MS^ *pro*^N.3P.+R.-A^ foi bom]

Interviewer: Fez o quê tanto ontem?

Participant: [^MS^ *pro*^N.3P.+R.+A^ foi bom]

Interviewer: Foi bom?

Participant: [^RP^ foi bom]

Interviewer: O que você fez?

Participant: [^MS.TS.+A^ ir no shopping]

Interviewer: Shopping. Que mais?

Participant: [^MS.TS.+A^ ir para o parque]

Interviewer: Ir para o parque. Que mais?

Participant: [^MS.TS.+A^ brincar]

Translation: - Then tell me how your day was yesterday - (It)^1^ was good. - What did you do so much yesterday? - (?)^2^ was good. - Was good? - (?)was good^3^. - What did you do? - To go^4^ to the mall. - The mall. What else? - To go^4^ to the park. – Go to the park. What else? – To^4^ play.

Comments: ^1^null 3Person non-anomalous pronouns; ^2^null 3Person anomalous pronoun, its referent is missing; ^3^phrase repetition; ^4^truncated anomalous sentence, ungrammatical use of the infinite verbal form.

*Waking narrative of a NSZ participant:*

Interviewer: Agora, conta para mim, o que você fez ontem.

Participant: [^MS^ eu^O.1P.+R.-A^ acordei] [^MS^ *pro*^N.1P.+R.-A^ fui direto para o computador] [^MS^ *pro*^N.1P.+R.-A^ fiquei 12 horas no computador] [^MS^ *pro*^N.1P.+R.-A^ saí] [^MS^ *pro*^N.1P.+R.-A^ fui comer] [^MS^ [^ES^ quando *pro*^N.1P.+R.-A^ terminei] *pro*^N.1P.+R.-A^ escovei os dentes] [^MS^ *pro*^N.1P.+R.-A^ tomei banho] [^MS^ *pro*^N.1P.+R.-A^ fui para o colégio] [^MS^ *pro*^N.1P.+R.-A^ passei o quinto horário] [^MS^ *pro*^N.3P.-R^ teve uma aula extra [^ES^ que foi com um dos professores [^ES.TS.-A^ que vieram]] [^MS^ *pro*^N.1P.+R.-A^ fui para casa] [^MS^ *pro*^N.1P.+R.-A^ cheguei] [^MS^ *pro*^N.1P.+R.-A^ arrumei o quarto]. [^MS^ *pro*^N.1P.+R.-A^ entrei no computador de novo] [^MS^ *pro*^N.1P.+R.-A^ saí] [^MS^ *pro*^N.1P.+R.-A^ andei de bicicleta] [^MS^ *pro*^N.1P.+R.-A^ voltei] [^MS^ *pro*^N.1P.+R.-A^ li um pedaço de um livro] [^MS^ e *pro*^N.1P.+R.-A^ fui dormir]

Translation: - Now, tell me what you did yesterday. - I woke up, (I) went straight to the computer. (I) stayed 12 hours at the computer. (I) left, (I) went to eat. When (I) finished, (I) brushed my teeth, (I) took a shower. (I) went to school. (I) passed the fifth hour. (There)^1^ was an extra class, which was with one of the professors that came (to school)^2^. (I) went home. (I) arrived, (I) cleaned the room. (I) entered the computer again. (I) left, (I) rode a bicycle. (I) returned. (I) read part of a book and (I) went to bed.

Comments: ^1^null 3Person non-referential (expletive) pronoun; ^2^truncated non-anomalous sentence, meaning is clear.
