# Supplementary material for: Grammatical impairment in schizophrenia: An exploratory study of the pronominal and sentential domains
Source: PLoS One. 2023 Sep 12;18(9):e0291446. doi: 10.1371/journal.pone.0291446 (PMC10497169; doi:10.1371/journal.pone.0291446)
Supplement: S2 Appendix — (DOCX) [file pone.0291446.s002.docx]

**S2 Appendix: Definition of the analyzed language variables, with examples.**

Language variables capture both the pronominal (1-8) and the sentential (9-12) domains. In the examples, *pro* was inserted in all occurrences of null pronouns. For more details see Materials and Methods.

| **Language variables** | **Definitions** | **Examples** |
| --- | --- | --- |
| 1. Overt Pronoun [O] | All subject pronouns with phonological content. | Eu entrei na sala.  *I entered in the room* |
| 2. Null Pronoun [N] | All subject pronouns without phonological content. | *pro* Entrei na sala.  *(I) entered in the room* |
| 3. Null 3P Referential Pronoun  [N3P+R] | All third person subject pronouns without phonological content and with definite interpretation. | *pro* Entrou na sala.  *(he/she) entered the room*  [*pro* has a specific well-defined referent] |
| 4. Null 3P Non-Referential Pronoun  [N3P-R] | All third person subject pronouns without phonological content and with impersonal, generic readings, and expletives. | *pro* Choveu muito.  *(It) rained a lot* |
| 5. Null 3P Referentially Anomalous Pronoun [N3P+R+A] | All third person subject pronouns without phonological content and without a clear, non-ambiguous antecedent/referent. | *pro* entrou na sala.  *(he/she) entered the room*  [*pro’s* antecedent cannot be recovered.] |
| 6. Null 3P Referentially Non-anomalous Pronoun [N3P+R-A] | All third person subject pronouns without phonological content and with a clear, non-ambiguous, readily recoverable antecedent/referent. | *pro* entrou na sala.  *(he/she) entered the room*  [*pro’s* antecedent can be recovered] |
| 7. Overt 3P Referentially Anomalous Pronoun [O3P+R+A] | All third person subject pronouns with phonological content, without a clear, non-ambiguous antecedent/referent. | Ela entrou na sala.  *(She) entered the room*  [The antecedent of *ela/she* cannot be recovered.] |
| 8. Overt 3P Referentially Non-anomalous Pronoun  [O3P+R-A] | All third person subject pronouns with phonological content and with a clear readily recoverable referent. | Ela entrou na sala.  *She entered the room*  [The antecedent of *ela/she* can be recovered.] |
| 9. Matrix Sentence [MS] | All syntactically independent sentences | Ela entrou na sala.  *She entered the room* |
| 10. Embedded Sentence [ES] | All sentences embedded under a predicate, whether the sentence be a complement or adjunct. | Eu acho que ela foi ontem.  *I think that she went yesterday*  *Eu cheguei* quando ela saiu.  I arrived when she left |
| 11. Truncated Anomalous Sentence [TS+A] | All cases of ungrammatical ellipsis. Incomplete ungrammatical sentences, where the meaning of missing part cannot be recovered from the linguistic context. | Eu tenho uma ligação muito grande.  *I have a very strong relation*  [The complement of the noun *ligação/relation* is missing and cannot be recovered.] |
| 12. Truncated Non-Anomalous Sentence [TS-A] | All cases of grammatical ellipsis. Incomplete grammatical sentences, with the meaning of the elided constituent being recoverable in the linguistic context. | Ele perguntava se eu estava bem, e eu dizia.  *He used to ask if I was fine, and I used to say (if I was fine)*  [The elided complement of the verb *dizer/to say* is recoverable] |
