# Supplementary material for: Grammatical impairment in schizophrenia: An exploratory study of the pronominal and sentential domains
Source: PLoS One. 2023 Sep 12;18(9):e0291446. doi: 10.1371/journal.pone.0291446 (PMC10497169; doi:10.1371/journal.pone.0291446)
Supplement: S3 Appendix — (DOCX) [file pone.0291446.s003.docx]

**S3 Appendix: Analyzed variables and final data of dream and waking narratives of samples 1 and 2.**

All analyzed data was obtained by dividing the number of occurrences of the target item by the narrative total number of words, and then multiplying it by 100. For more details see Materials and Methods.

**S3 Table 1:** Analyzed variables.

| **Description of analyzed variables** | |
| --- | --- |
| **CT** | Control Participant |
| **SZ** | Participant with schizophrenia |
| **WC** | Word Count, i.e., total number of words of the narrative |
| **Overt** | Ratio of Overt Pronouns in the Narrative |
| **Null** | Ratio of Null Pronouns in the Narrative |
| **N3P+R** | Ratio of Null Third Person Referential Pronouns in the Narrative |
| **N3P-R** | Ratio of Null Third Person Non-referential Pronouns in the Narrative |
| **N3PR+A** | Ratio of Null Third Person Referentially Anomalous Pronouns in the Narrative |
| **N3PR-A** | Ratio of Null Third Person Referentially Non-anomalous Pronouns in the Narrative |
| **O3PR+A** | Ratio of Overt Third Person Referentially Anomalous Pronouns in the Narrative |
| **O3PR-A** | Ratio of Overt Third Person Referentially Non-anomalous Pronouns in the Narrative |
| **MS** | Ratio of Matrix Sentences in the Narrative |
| **ES** | Ratio of Embedded Sentences in the Narrative |
| **TS+A** | Ratio of Truncated Sentences Anomalous in the Narrative |
| **TS-A** | Ratio of Truncated Sentences Non-anomalous in the Narrative |

**S3 Table 2:** Sample 1 dream narrative data.

| **Sample 1: Dream Data** | | | | | | | | | | | | | | |
| --- | --- | --- | --- | --- | --- | --- | --- | --- | --- | --- | --- | --- | --- | --- |
| **Participant #** | **Group** | **WC** | **Overt** | **Null** | **N3P+R** | **N3P-R** | **N3PR+A** | **N3PR-AN** | **O3PR+A** | **O3PR-A** | **MS** | **ES** | **TS+A** | **TS-A** |
| 1 | CT | 275 | 9,09 | 5,82 | 2,18 | 0,36 | 0,36 | 1,82 | 0,00 | 0,00 | 10,18 | 7,27 | 1,09 | 0,00 |
| 2 | CT | 388 | 10,31 | 8,25 | 2,32 | 4,12 | 0,00 | 2,32 | 0,00 | 2,32 | 16,24 | 7,22 | 1,55 | 0,52 |
| 3 | CT | 122 | 9,84 | 6,56 | 0,00 | 2,46 | 0,00 | 0,00 | 0,00 | 0,00 | 13,93 | 3,28 | 0,82 | 0,82 |
| 4 | CT | 175 | 5,71 | 9,14 | 3,43 | 2,86 | 1,71 | 1,71 | 5,71 | 0,00 | 12,57 | 5,14 | 1,14 | 0,00 |
| 5 | CT | 228 | 6,58 | 8,77 | 2,19 | 2,63 | 0,88 | 1,32 | 0,00 | 0,88 | 16,23 | 4,82 | 0,44 | 0,00 |
| 6 | CT | 260 | 4,23 | 8,08 | 2,69 | 2,31 | 0,77 | 1,92 | 0,00 | 1,92 | 13,08 | 6,15 | 1,54 | 0,00 |
| 7 | CT | 438 | 11,19 | 4,34 | 1,37 | 0,91 | 0,00 | 1,37 | 0,23 | 2,51 | 15,75 | 7,08 | 0,46 | 0,23 |
| 8 | CT | 210 | 6,19 | 4,29 | 0,00 | 4,29 | 0,00 | 0,00 | 0,00 | 0,48 | 9,52 | 10,00 | 0,95 | 0,00 |
| 9 | CT | 421 | 10,21 | 5,23 | 1,43 | 2,38 | 0,24 | 1,19 | 0,00 | 0,48 | 11,88 | 7,36 | 1,90 | 0,71 |
| 10 | CT | 160 | 9,38 | 9,38 | 3,13 | 1,88 | 0,00 | 3,13 | 0,00 | 0,00 | 15,00 | 6,25 | 0,63 | 0,00 |
| 11 | CT | 556 | 11,33 | 4,68 | 1,08 | 0,72 | 0,72 | 0,36 | 0,00 | 1,44 | 13,31 | 7,91 | 0,90 | 0,36 |
| 12 | CT | 276 | 6,88 | 3,62 | 1,81 | 1,09 | 0,36 | 1,45 | 1,45 | 2,17 | 11,23 | 9,78 | 0,72 | 1,45 |
| 13 | CT | 121 | 9,09 | 6,61 | 1,65 | 2,48 | 0,83 | 0,83 | 0,00 | 1,65 | 12,40 | 10,74 | 0,00 | 0,83 |
| 14 | CT | 746 | 6,17 | 4,83 | 1,34 | 0,54 | 0,80 | 0,54 | 0,00 | 0,94 | 12,06 | 6,70 | 0,94 | 0,54 |
| 15 | CT | 100 | 7,00 | 13,00 | 3,00 | 3,00 | 1,00 | 2,00 | 0,00 | 1,00 | 19,00 | 3,00 | 4,00 | 0,00 |
| 16 | CT | 189 | 6,88 | 8,99 | 4,23 | 0,53 | 2,12 | 2,12 | 0,53 | 1,59 | 13,76 | 5,29 | 0,00 | 0,00 |
| 17 | CT | 204 | 6,86 | 8,82 | 3,43 | 0,98 | 0,00 | 3,43 | 0,00 | 0,98 | 16,18 | 6,37 | 0,98 | 0,98 |
| 18 | CT | 406 | 8,62 | 6,90 | 2,71 | 1,72 | 0,74 | 1,97 | 0,00 | 0,25 | 13,79 | 7,64 | 0,49 | 0,49 |
| 19 | CT | 157 | 4,46 | 10,83 | 2,55 | 0,64 | 0,00 | 2,55 | 0,00 | 0,00 | 15,29 | 5,73 | 0,00 | 0,64 |
| 20 | CT | 297 | 8,08 | 7,41 | 0,34 | 4,71 | 0,00 | 0,34 | 0,00 | 0,34 | 13,47 | 8,42 | 0,34 | 0,00 |
| 21 | SZ | 30 | 3,33 | 26,67 | 6,67 | 0,00 | 0,00 | 6,67 | 0,00 | 0,00 | 23,33 | 3,33 | 0,00 | 0,00 |
| 22 | SZ | 243 | 5,35 | 11,52 | 5,35 | 3,29 | 3,70 | 1,65 | 0,82 | 0,00 | 13,99 | 8,23 | 3,29 | 0,00 |
| 23 | SZ | 91 | 9,89 | 9,89 | 0,00 | 7,69 | 0,00 | 0,00 | 1,10 | 0,00 | 12,09 | 14,29 | 2,20 | 0,00 |
| 24 | SZ | 81 | 4,94 | 13,58 | 3,70 | 3,70 | 0,00 | 3,70 | 0,00 | 0,00 | 17,28 | 7,41 | 1,23 | 0,00 |
| 25 | SZ | 95 | 6,32 | 10,53 | 1,05 | 1,05 | 1,05 | 0,00 | 1,05 | 0,00 | 18,95 | 6,32 | 0,00 | 0,00 |
| 26 | SZ | 93 | 7,53 | 7,53 | 2,15 | 1,08 | 1,08 | 1,08 | 0,00 | 0,00 | 15,05 | 2,15 | 3,23 | 0,00 |
| 27 | SZ | 198 | 5,56 | 10,61 | 3,03 | 2,53 | 1,52 | 1,52 | 0,51 | 0,00 | 14,65 | 6,57 | 0,51 | 0,00 |
| 28 | SZ | 123 | 13,82 | 9,76 | 2,44 | 1,63 | 1,63 | 0,81 | 0,81 | 0,00 | 21,14 | 4,88 | 4,07 | 0,00 |
| 29 | SZ | 100 | 10,00 | 16,00 | 8,00 | 1,00 | 0,00 | 8,00 | 0,00 | 5,00 | 20,00 | 9,00 | 1,00 | 0,00 |
| 30 | SZ | 102 | 3,92 | 12,75 | 0,00 | 0,00 | 0,00 | 0,00 | 0,00 | 0,98 | 16,67 | 0,98 | 0,98 | 0,00 |
| 31 | SZ | 117 | 6,84 | 5,13 | 1,71 | 2,56 | 0,00 | 1,71 | 0,00 | 0,00 | 12,82 | 5,98 | 1,71 | 0,85 |
| 32 | SZ | 384 | 10,94 | 5,99 | 2,08 | 0,78 | 1,04 | 1,04 | 0,00 | 2,60 | 16,41 | 7,03 | 0,26 | 0,26 |
| 33 | SZ | 107 | 7,48 | 7,48 | 1,87 | 1,87 | 0,00 | 1,87 | 0,00 | 0,93 | 14,95 | 9,35 | 0,93 | 0,00 |
| 34 | SZ | 84 | 9,52 | 5,95 | 0,00 | 1,19 | 0,00 | 0,00 | 0,00 | 1,19 | 14,29 | 3,57 | 0,00 | 0,00 |
| 35 | SZ | 64 | 4,69 | 1,56 | 0,00 | 0,00 | 0,00 | 0,00 | 0,00 | 3,13 | 14,06 | 9,38 | 0,00 | 0,00 |
| 36 | SZ | 39 | 7,69 | 10,26 | 2,56 | 7,69 | 0,00 | 2,56 | 0,00 | 0,00 | 15,38 | 2,56 | 0,00 | 0,00 |
| 37 | SZ | 58 | 5,17 | 13,79 | 3,45 | 3,45 | 3,45 | 0,00 | 0,00 | 0,00 | 18,97 | 6,90 | 3,45 | 0,00 |
| 38 | SZ | 206 | 10,68 | 8,25 | 2,43 | 1,94 | 0,97 | 1,46 | 2,43 | 2,43 | 16,99 | 5,34 | 2,43 | 0,00 |
| 39 | SZ | 281 | 6,76 | 11,03 | 5,34 | 1,78 | 3,56 | 1,78 | 1,07 | 0,00 | 16,01 | 8,19 | 2,14 | 0,00 |
| 40 | SZ | 85 | 3,53 | 4,71 | 1,18 | 0,00 | 0,00 | 1,18 | 0,00 | 1,18 | 20,00 | 5,88 | 1,18 | 0,00 |

**S3 Table 3:** Sample 1 waking narrative data.

| **Sample 1: Waking Data** | | | | | | | | | | | | | | |
| --- | --- | --- | --- | --- | --- | --- | --- | --- | --- | --- | --- | --- | --- | --- |
| **Participant #** | **Group** | **WC** | **Overt** | **Null** | **N3P+R** | **N3P-R** | **N3PR+A** | **N3PR-AN** | **O3PR+A** | **O3PR-A** | **MS** | **ES** | **TS+A** | **TS-A** |
| 1 | CT | 188 | 2,13 | 10,11 | 0,53 | 1,06 | 0,00 | 0,53 | 0,00 | 1,06 | 15,43 | 1,06 | 0,00 | 1,06 |
| 2 | CT | 160 | 6,25 | 15,00 | 1,88 | 2,50 | 1,25 | 0,63 | 0,00 | 0,00 | 18,13 | 8,75 | 1,88 | 0,00 |
| 3 | CT | 21 | 0,00 | 0,00 | 0,00 | 0,00 | 0,00 | 0,00 | 0,00 | 0,00 | 4,76 | 4,76 | 0,00 | 0,00 |
| 4 | CT | 58 | 1,72 | 17,24 | 0,00 | 1,72 | 0,00 | 0,00 | 0,00 | 0,00 | 24,14 | 1,72 | 5,17 | 0,00 |
| 5 | CT | 95 | 7,37 | 9,47 | 2,11 | 0,00 | 1,05 | 1,05 | 0,00 | 2,11 | 13,68 | 7,37 | 0,00 | 1,05 |
| 6 | CT | 96 | 8,33 | 9,38 | 1,04 | 0,00 | 0,00 | 1,04 | 3,13 | 1,04 | 18,75 | 2,08 | 2,08 | 0,00 |
| 7 | CT | 282 | 6,74 | 4,61 | 1,42 | 0,00 | 0,35 | 1,06 | 0,00 | 1,42 | 12,06 | 6,03 | 0,35 | 0,00 |
| 8 | CT | 57 | 5,26 | 10,53 | 0,00 | 1,75 | 0,00 | 0,00 | 0,00 | 0,00 | 15,79 | 7,02 | 0,00 | 0,00 |
| 9 | CT | 394 | 9,14 | 4,57 | 1,02 | 1,02 | 0,51 | 0,51 | 0,00 | 0,00 | 11,42 | 10,91 | 1,52 | 0,00 |
| 10 | CT | 69 | 13,04 | 8,70 | 0,00 | 0,00 | 0,00 | 0,00 | 1,45 | 0,00 | 18,84 | 7,25 | 4,35 | 0,00 |
| 11 | CT | 246 | 6,91 | 6,50 | 0,81 | 0,41 | 0,41 | 0,41 | 0,00 | 0,00 | 15,45 | 4,07 | 0,41 | 0,81 |
| 12 | CT | 127 | 8,66 | 9,45 | 0,00 | 1,57 | 0,00 | 0,00 | 0,00 | 0,00 | 14,17 | 7,87 | 0,00 | 0,00 |
| 13 | CT | 108 | 6,48 | 5,56 | 0,93 | 0,00 | 0,00 | 0,93 | 0,00 | 1,85 | 12,04 | 2,78 | 1,85 | 0,00 |
| 14 | CT | 130 | 9,23 | 9,23 | 4,62 | 0,00 | 0,00 | 4,62 | 2,31 | 1,54 | 16,92 | 3,85 | 2,31 | 0,00 |
| 15 | CT | 45 | 0,00 | 11,11 | 0,00 | 2,22 | 0,00 | 0,00 | 0,00 | 0,00 | 15,56 | 2,22 | 0,00 | 2,22 |
| 16 | CT | 78 | 6,41 | 14,10 | 1,28 | 0,00 | 1,28 | 0,00 | 0,00 | 2,56 | 25,64 | 2,56 | 0,00 | 0,00 |
| 17 | CT | 404 | 9,41 | 7,43 | 1,24 | 1,98 | 0,00 | 1,24 | 0,00 | 1,98 | 16,09 | 4,70 | 1,24 | 0,00 |
| 18 | CT | 20 | 0,00 | 35,00 | 0,00 | 0,00 | 0,00 | 0,00 | 0,00 | 0,00 | 35,00 | 0,00 | 0,00 | 0,00 |
| 19 | CT | 14 | 0,00 | 14,29 | 0,00 | 0,00 | 0,00 | 0,00 | 0,00 | 0,00 | 14,29 | 0,00 | 0,00 | 0,00 |
| 20 | CT | 32 | 6,25 | 18,75 | 0,00 | 0,00 | 0,00 | 0,00 | 0,00 | 0,00 | 18,75 | 9,38 | 0,00 | 0,00 |
| 21 | SZ | 3 | 0,00 | 33,33 | 0,00 | 0,00 | 0,00 | 0,00 | 0,00 | 0,00 | 33,33 | 0,00 | 0,00 | 0,00 |
| 22 | SZ | 138 | 9,42 | 10,14 | 5,07 | 0,72 | 0,00 | 5,07 | 0,00 | 1,45 | 18,84 | 5,07 | 2,17 | 0,72 |
| 23 | SZ | 49 | 8,16 | 4,08 | 0,00 | 4,08 | 0,00 | 0,00 | 0,00 | 0,00 | 20,41 | 2,04 | 0,00 | 0,00 |
| 24 | SZ | 86 | 11,63 | 9,30 | 2,33 | 3,49 | 1,16 | 1,16 | 0,00 | 1,16 | 12,79 | 8,14 | 1,16 | 0,00 |
| 25 | SZ | 129 | 4,65 | 10,85 | 2,33 | 0,78 | 0,00 | 2,33 | 0,00 | 0,78 | 17,05 | 4,65 | 0,00 | 0,00 |
| 26 | SZ | 249 | 8,84 | 10,44 | 4,02 | 1,20 | 4,02 | 0,00 | 0,00 | 0,80 | 17,67 | 6,43 | 2,81 | 0,40 |
| 27 | SZ | 129 | 6,20 | 6,98 | 1,55 | 1,55 | 0,00 | 1,55 | 0,00 | 0,00 | 16,28 | 6,20 | 0,00 | 2,33 |
| 28 | SZ | 127 | 7,87 | 9,45 | 4,72 | 0,79 | 3,94 | 0,79 | 2,36 | 0,00 | 17,32 | 5,51 | 0,79 | 0,00 |
| 29 | SZ | 27 | 3,70 | 14,81 | 11,11 | 0,00 | 0,00 | 11,11 | 0,00 | 0,00 | 22,22 | 3,70 | 0,00 | 0,00 |
| 30 | SZ | 56 | 3,57 | 14,29 | 1,79 | 0,00 | 0,00 | 1,79 | 0,00 | 0,00 | 19,64 | 1,79 | 0,00 | 0,00 |
| 31 | SZ | 219 | 6,39 | 9,59 | 0,91 | 1,83 | 0,91 | 0,00 | 0,46 | 0,00 | 17,81 | 4,57 | 3,20 | 0,46 |
| 32 | SZ | 170 | 5,29 | 8,82 | 0,59 | 1,76 | 0,00 | 0,59 | 0,00 | 0,59 | 14,12 | 8,24 | 0,00 | 0,00 |
| 33 | SZ | 95 | 0,00 | 17,89 | 3,16 | 0,00 | 0,00 | 3,16 | 0,00 | 0,00 | 16,84 | 2,11 | 1,05 | 0,00 |
| 34 | SZ | 48 | 10,42 | 12,50 | 0,00 | 0,00 | 0,00 | 0,00 | 2,08 | 2,08 | 20,83 | 4,17 | 0,00 | 0,00 |
| 35 | SZ | 84 | 5,95 | 10,71 | 0,00 | 1,19 | 0,00 | 0,00 | 0,00 | 2,38 | 21,43 | 5,95 | 1,19 | 2,38 |
| 36 | SZ | 20 | 0,00 | 20,00 | 15,00 | 0,00 | 5,00 | 10,00 | 0,00 | 0,00 | 15,00 | 0,00 | 0,00 | 0,00 |
| 37 | SZ | 72 | 1,39 | 13,89 | 5,56 | 1,39 | 1,39 | 4,17 | 0,00 | 0,00 | 16,67 | 6,94 | 1,39 | 0,00 |
| 38 | SZ | 107 | 7,48 | 7,48 | 0,93 | 4,67 | 0,00 | 0,93 | 1,87 | 0,93 | 12,15 | 14,95 | 3,74 | 0,00 |
| 39 | SZ | 111 | 7,21 | 5,41 | 2,70 | 0,90 | 0,90 | 1,80 | 0,90 | 4,50 | 12,61 | 8,11 | 0,00 | 0,90 |
| 40 | SZ | 33 | 6,06 | 15,15 | 3,03 | 0,00 | 0,00 | 3,03 | 0,00 | 0,00 | 21,21 | 3,03 | 3,03 | 0,00 |

**S3 Table 4:** Sample 2 dream narratives data.

| **Sample 2: Dream Data** | | | | | | | | | | | | | | |
| --- | --- | --- | --- | --- | --- | --- | --- | --- | --- | --- | --- | --- | --- | --- |
| **Participant #** | **Group** | **WC** | **Overt** | **Null** | **N3P+R** | **N3P-R** | **N3PR+A** | **N3PR-AN** | **O3PR+A** | **O3PR-A** | **MS** | **ES** | **TS+A** | **TS-A** |
| 1 | CT | 97 | 4,12 | 8,25 | 4,12 | 1,03 | 0,00 | 4,12 | 0,00 | 1,03 | 16,49 | 8,25 | 0,0 | 0,0 |
| 2 | CT | 88 | 9,09 | 7,95 | 4,55 | 0,00 | 0,00 | 4,55 | 0,00 | 1,14 | 20,45 | 4,55 | 1,1 | 0,0 |
| 3 | CT | 95 | 8,42 | 5,26 | 1,05 | 2,11 | 0,00 | 1,05 | 0,00 | 1,05 | 11,58 | 9,47 | 0,0 | 0,0 |
| 4 | CT | 56 | 16,07 | 1,79 | 0,00 | 0,00 | 0,00 | 0,00 | 0,00 | 3,57 | 16,07 | 8,93 | 1,8 | 0,0 |
| 5 | CT | 46 | 8,70 | 6,52 | 0,00 | 0,00 | 0,00 | 0,00 | 0,00 | 0,00 | 17,39 | 2,17 | 0,0 | 0,0 |
| 6 | CT | 68 | 11,76 | 4,41 | 2,94 | 1,47 | 0,00 | 2,94 | 0,00 | 4,41 | 19,12 | 4,41 | 0,0 | 1,5 |
| 7 | CT | 27 | 11,11 | 7,41 | 7,41 | 0,00 | 0,00 | 7,41 | 0,00 | 7,41 | 18,52 | 0,00 | 0,0 | 0,0 |
| 8 | CT | 76 | 7,89 | 3,95 | 1,32 | 1,32 | 0,00 | 1,32 | 0,00 | 0,00 | 9,21 | 9,21 | 1,3 | 0,0 |
| 9 | CT | 59 | 5,08 | 3,39 | 1,69 | 1,69 | 0,00 | 1,69 | 0,00 | 3,39 | 16,95 | 8,47 | 1,7 | 0,0 |
| 10 | CT | 52 | 3,85 | 5,77 | 1,92 | 1,92 | 0,00 | 1,92 | 0,00 | 1,92 | 13,46 | 5,77 | 0,0 | 1,9 |
| 11 | CT | 33 | 3,03 | 15,15 | 0,00 | 0,00 | 0,00 | 0,00 | 0,00 | 0,00 | 27,27 | 3,03 | 3,0 | 0,0 |
| 12 | CT | 28 | 10,71 | 10,71 | 0,00 | 10,71 | 0,00 | 0,00 | 0,00 | 3,57 | 21,43 | 3,57 | 0,0 | 0,0 |
| 13 | CT | 48 | 6,25 | 8,33 | 0,00 | 4,17 | 0,00 | 0,00 | 0,00 | 0,00 | 16,67 | 6,25 | 0,0 | 0,0 |
| 14 | CT | 26 | 3,85 | 0,00 | 0,00 | 0,00 | 0,00 | 0,00 | 0,00 | 0,00 | 7,69 | 0,00 | 0,0 | 3,8 |
| 15 | CT | 80 | 6,25 | 5,00 | 2,50 | 1,25 | 0,00 | 2,50 | 0,00 | 1,25 | 11,25 | 8,75 | 0,0 | 0,0 |
| 16 | CT | 77 | 2,60 | 6,49 | 0,00 | 0,00 | 0,00 | 0,00 | 1,30 | 0,00 | 19,48 | 6,49 | 1,3 | 1,3 |
| 17 | CT | 97 | 9,28 | 3,09 | 0,00 | 1,03 | 0,00 | 0,00 | 0,00 | 0,00 | 11,34 | 6,19 | 1,0 | 0,0 |
| 18 | CT | 65 | 9,23 | 12,31 | 0,00 | 1,54 | 0,00 | 0,00 | 0,00 | 0,00 | 15,38 | 10,77 | 0,0 | 1,5 |
| 19 | CT | 57 | 5,26 | 7,02 | 3,51 | 0,00 | 0,00 | 3,51 | 0,00 | 0,00 | 17,54 | 7,02 | 0,0 | 0,0 |
| 20 | CT | 60 | 10,00 | 10,00 | 3,33 | 1,67 | 0,00 | 3,33 | 0,00 | 0,00 | 16,67 | 6,67 | 0,0 | 0,0 |
| 21 | SZ | 29 | 0,00 | 13,79 | 3,45 | 0,00 | 0,00 | 3,45 | 0,00 | 0,00 | 20,69 | 6,90 | 3,4 | 0,0 |
| 22 | SZ | 26 | 11,54 | 0,00 | 0,00 | 0,00 | 0,00 | 0,00 | 0,00 | 0,00 | 7,69 | 7,69 | 0,0 | 0,0 |
| 23 | SZ | 44 | 6,82 | 11,36 | 0,00 | 0,00 | 0,00 | 0,00 | 0,00 | 0,00 | 18,18 | 0,00 | 0,0 | 0,0 |
| 24 | SZ | 41 | 4,88 | 14,63 | 2,44 | 2,44 | 0,00 | 2,44 | 0,00 | 0,00 | 17,07 | 7,32 | 0,0 | 2,4 |
| 25 | SZ | 32 | 3,13 | 0,00 | 0,00 | 0,00 | 0,00 | 0,00 | 0,00 | 0,00 | 31,25 | 3,13 | 31,3 | 0,0 |
| 26 | SZ | 15 | 0,00 | 13,33 | 6,67 | 0,00 | 6,67 | 0,00 | 0,00 | 0,00 | 13,33 | 0,00 | 6,7 | 0,0 |
| 27 | SZ | 21 | 14,29 | 4,76 | 0,00 | 0,00 | 0,00 | 0,00 | 0,00 | 0,00 | 14,29 | 4,76 | 0,0 | 0,0 |
| 28 | SZ | 24 | 12,50 | 4,17 | 0,00 | 0,00 | 0,00 | 0,00 | 0,00 | 4,17 | 16,67 | 0,00 | 0,0 | 0,0 |
| 29 | SZ | 30 | 10,00 | 6,67 | 6,67 | 0,00 | 0,00 | 6,67 | 0,00 | 0,00 | 16,67 | 3,33 | 0,0 | 0,0 |
| 30 | SZ | 43 | 11,63 | 9,30 | 2,33 | 2,33 | 0,00 | 2,33 | 0,00 | 0,00 | 16,28 | 6,98 | 0,0 | 0,0 |
| 31 | SZ | 108 | 6,48 | 8,33 | 3,70 | 0,00 | 0,00 | 3,70 | 0,00 | 0,00 | 15,74 | 7,41 | 0,9 | 0,0 |
| 1 | CT | 97 | 4,12 | 8,25 | 4,12 | 1,03 | 0,00 | 4,12 | 0,00 | 1,03 | 16,49 | 8,25 | 0,0 | 0,0 |
| 2 | CT | 88 | 9,09 | 7,95 | 4,55 | 0,00 | 0,00 | 4,55 | 0,00 | 1,14 | 20,45 | 4,55 | 1,1 | 0,0 |
| 3 | CT | 95 | 8,42 | 5,26 | 1,05 | 2,11 | 0,00 | 1,05 | 0,00 | 1,05 | 11,58 | 9,47 | 0,0 | 0,0 |
| 4 | CT | 56 | 16,07 | 1,79 | 0,00 | 0,00 | 0,00 | 0,00 | 0,00 | 3,57 | 16,07 | 8,93 | 1,8 | 0,0 |
| 5 | CT | 46 | 8,70 | 6,52 | 0,00 | 0,00 | 0,00 | 0,00 | 0,00 | 0,00 | 17,39 | 2,17 | 0,0 | 0,0 |
| 6 | CT | 68 | 11,76 | 4,41 | 2,94 | 1,47 | 0,00 | 2,94 | 0,00 | 4,41 | 19,12 | 4,41 | 0,0 | 1,5 |
| 7 | CT | 27 | 11,11 | 7,41 | 7,41 | 0,00 | 0,00 | 7,41 | 0,00 | 7,41 | 18,52 | 0,00 | 0,0 | 0,0 |
| 8 | CT | 76 | 7,89 | 3,95 | 1,32 | 1,32 | 0,00 | 1,32 | 0,00 | 0,00 | 9,21 | 9,21 | 1,3 | 0,0 |
| 9 | CT | 59 | 5,08 | 3,39 | 1,69 | 1,69 | 0,00 | 1,69 | 0,00 | 3,39 | 16,95 | 8,47 | 1,7 | 0,0 |

**S3 Table 5:** Sample 2 waking narratives data.

| **Sample 2: Waking Data** | | | | | | | | | | | | | | |
| --- | --- | --- | --- | --- | --- | --- | --- | --- | --- | --- | --- | --- | --- | --- |
| **Participant #** | **Group** | **WC** | **Overt** | **Null** | **N3P+R** | **N3P-R** | **N3PR+A** | **N3PR-AN** | **O3PR+A** | **O3PR-A** | **MS** | **ES** | **TS+A** | **TS-A** |
| 1 | CT | 71 | 7,04 | 7,04 | 0,0 | 1,4 | 0,0 | 0,0 | 0,0 | 0,0 | 18,31 | 4,23 | 0,0 | 0,0 |
| 2 | CT | 40 | 15,00 | 5,00 | 0,0 | 2,5 | 0,0 | 0,0 | 0,0 | 0,0 | 20,00 | 0,00 | 0,0 | 0,0 |
| 3 | CT | 82 | 9,76 | 2,44 | 0,0 | 0,0 | 0,0 | 0,0 | 0,0 | 0,0 | 10,98 | 9,76 | 0,0 | 0,0 |
| 4 | CT | 37 | 5,41 | 8,11 | 0,0 | 0,0 | 0,0 | 0,0 | 0,0 | 0,0 | 16,22 | 5,41 | 2,7 | 0,0 |
| 5 | CT | 15 | 0,00 | 46,67 | 0,0 | 0,0 | 0,0 | 0,0 | 0,0 | 0,0 | 46,67 | 0,00 | 0,0 | 0,0 |
| 6 | CT | 66 | 6,06 | 19,70 | 0,0 | 3,0 | 0,0 | 0,0 | 0,0 | 0,0 | 25,76 | 3,03 | 3,0 | 0,0 |
| 7 | CT | 50 | 4,00 | 16,00 | 0,0 | 0,0 | 0,0 | 0,0 | 0,0 | 0,0 | 16,00 | 4,00 | 0,0 | 0,0 |
| 8 | CT | 70 | 1,43 | 17,14 | 0,0 | 0,0 | 0,0 | 0,0 | 0,0 | 0,0 | 17,14 | 2,86 | 0,0 | 0,0 |
| 9 | CT | 55 | 9,09 | 10,91 | 0,0 | 1,8 | 0,0 | 0,0 | 0,0 | 1,8 | 18,18 | 3,64 | 0,0 | 0,0 |
| 10 | CT | 57 | 5,26 | 8,77 | 1,8 | 0,0 | 0,0 | 1,8 | 0,0 | 0,0 | 17,54 | 3,51 | 1,8 | 0,0 |
| 11 | CT | 46 | 8,70 | 8,70 | 4,3 | 2,2 | 2,2 | 2,2 | 0,0 | 0,0 | 13,04 | 6,52 | 0,0 | 0,0 |
| 12 | CT | 25 | 8,00 | 20,00 | 0,0 | 0,0 | 0,0 | 0,0 | 0,0 | 4,0 | 32,00 | 0,00 | 4,0 | 0,0 |
| 13 | CT | 28 | 3,57 | 0,00 | 0,0 | 0,0 | 0,0 | 0,0 | 0,0 | 0,0 | 3,57 | 0,00 | 0,0 | 0,0 |
| 14 | CT | 41 | 0,00 | 26,83 | 0,0 | 0,0 | 0,0 | 0,0 | 0,0 | 0,0 | 26,83 | 2,44 | 0,0 | 0,0 |
| 15 | CT | 46 | 8,70 | 6,52 | 0,0 | 0,0 | 0,0 | 0,0 | 0,0 | 0,0 | 19,57 | 2,17 | 0,0 | 0,0 |
| 16 | CT | 38 | 0,00 | 21,05 | 0,0 | 0,0 | 0,0 | 0,0 | 0,0 | 0,0 | 23,68 | 0,00 | 0,0 | 2,6 |
| 17 | CT | 63 | 7,94 | 14,29 | 0,0 | 0,0 | 0,0 | 0,0 | 0,0 | 0,0 | 22,22 | 1,59 | 0,0 | 0,0 |
| 18 | CT | 72 | 1,39 | 26,39 | 0,0 | 1,4 | 0,0 | 0,0 | 0,0 | 0,0 | 26,39 | 4,17 | 0,0 | 0,0 |
| 19 | CT | 49 | 4,08 | 28,57 | 0,0 | 4,1 | 0,0 | 0,0 | 0,0 | 0,0 | 30,61 | 2,04 | 0,0 | 0,0 |
| 20 | CT | 42 | 2,38 | 23,81 | 0,0 | 0,0 | 0,0 | 0,0 | 0,0 | 0,0 | 26,19 | 2,38 | 0,0 | 0,0 |
| 21 | SZ | 44 | 6,82 | 11,36 | 0,0 | 2,3 | 0,0 | 0,0 | 0,0 | 0,0 | 18,18 | 6,82 | 2,3 | 0,0 |
| 22 | SZ | 39 | 7,69 | 5,13 | 0,0 | 0,0 | 0,0 | 0,0 | 0,0 | 0,0 | 12,82 | 0,00 | 0,0 | 0,0 |
| 23 | SZ | 51 | 3,92 | 9,80 | 0,0 | 3,9 | 0,0 | 0,0 | 0,0 | 0,0 | 19,61 | 1,96 | 2,0 | 0,0 |
| 24 | SZ | 66 | 3,03 | 15,15 | 4,5 | 0,0 | 4,5 | 0,0 | 0,0 | 0,0 | 21,21 | 4,55 | 0,0 | 0,0 |
| 25 | SZ | 36 | 11,11 | 13,89 | 2,8 | 0,0 | 0,0 | 2,8 | 0,0 | 0,0 | 25,00 | 0,00 | 2,8 | 0,0 |
| 26 | SZ | 14 | 0,00 | 14,29 | 14,3 | 0,0 | 7,1 | 7,1 | 0,0 | 0,0 | 35,71 | 0,00 | 21,4 | 0,0 |
| 27 | SZ | 21 | 0,00 | 14,29 | 0,0 | 0,0 | 0,0 | 0,0 | 0,0 | 0,0 | 28,57 | 0,00 | 0,0 | 0,0 |
| 28 | SZ | 28 | 3,57 | 14,29 | 3,6 | 0,0 | 0,0 | 3,6 | 0,0 | 0,0 | 21,43 | 0,00 | 0,0 | 0,0 |
| 29 | SZ | 24 | 16,67 | 4,17 | 4,2 | 0,0 | 4,2 | 0,0 | 0,0 | 0,0 | 16,67 | 4,17 | 0,0 | 0,0 |
| 30 | SZ | 35 | 5,71 | 2,86 | 0,0 | 2,9 | 0,0 | 0,0 | 0,0 | 0,0 | 11,43 | 8,57 | 2,9 | 2,9 |
| 31 | SZ | 22 | 0,00 | 18,18 | 4,5 | 4,5 | 0,0 | 4,5 | 0,0 | 0,0 | 18,18 | 0,00 | 0,0 | 0,0 |
| 1 | CT | 71 | 7,04 | 7,04 | 0,0 | 1,4 | 0,0 | 0,0 | 0,0 | 0,0 | 18,31 | 4,23 | 0,0 | 0,0 |
| 2 | CT | 40 | 15,00 | 5,00 | 0,0 | 2,5 | 0,0 | 0,0 | 0,0 | 0,0 | 20,00 | 0,00 | 0,0 | 0,0 |
| 3 | CT | 82 | 9,76 | 2,44 | 0,0 | 0,0 | 0,0 | 0,0 | 0,0 | 0,0 | 10,98 | 9,76 | 0,0 | 0,0 |
| 4 | CT | 37 | 5,41 | 8,11 | 0,0 | 0,0 | 0,0 | 0,0 | 0,0 | 0,0 | 16,22 | 5,41 | 2,7 | 0,0 |
| 5 | CT | 15 | 0,00 | 46,67 | 0,0 | 0,0 | 0,0 | 0,0 | 0,0 | 0,0 | 46,67 | 0,00 | 0,0 | 0,0 |
| 6 | CT | 66 | 6,06 | 19,70 | 0,0 | 3,0 | 0,0 | 0,0 | 0,0 | 0,0 | 25,76 | 3,03 | 3,0 | 0,0 |
| 7 | CT | 50 | 4,00 | 16,00 | 0,0 | 0,0 | 0,0 | 0,0 | 0,0 | 0,0 | 16,00 | 4,00 | 0,0 | 0,0 |
| 8 | CT | 70 | 1,43 | 17,14 | 0,0 | 0,0 | 0,0 | 0,0 | 0,0 | 0,0 | 17,14 | 2,86 | 0,0 | 0,0 |
| 9 | CT | 55 | 9,09 | 10,91 | 0,0 | 1,8 | 0,0 | 0,0 | 0,0 | 1,8 | 18,18 | 3,64 | 0,0 | 0,0 |
