# Supplementary material for: Grammatical impairment in schizophrenia: An exploratory study of the pronominal and sentential domains
Source: PLoS One. 2023 Sep 12;18(9):e0291446. doi: 10.1371/journal.pone.0291446 (PMC10497169; doi:10.1371/journal.pone.0291446)
Supplement: S4 Appendix — (DOCX) [file pone.0291446.s004.docx]

**S4 Appendix: Full regression data.**

Data from multiple correlation analyses conducted to determine the presence of confounding effect of age and education on the correlations presented on Table 6 of the manuscript. For more details see Stage 2: correlation analysis.

**S4 Table 1:** Raw data from sample 1 participants: linguistic variables with significant group differences in DM and WK, the scores of the psychometric scales, age and years of education.

| MS | TS-A | Null | 3NP+R | PANSS | | | Age | Education |
| --- | --- | --- | --- | --- | --- | --- | --- | --- |
|  |  |  |  | Total | Positive | Negative |  |  |
| 7,27 | 0,00 | 5,82 | 0,53 | 33 | 10 | 7 | 32 | 12 |
| 12,89 | 0,52 | 8,25 | 1,88 | 35 | 10 | 7 | 18 | 6 |
| 13,93 | 0,82 | 6,56 | 0,00 | 38 | 7 | 7 | 23 | 6 |
| 10,29 | 0,00 | 9,14 | 0,00 | 31 | 7 | 8 | 41 | 1 |
| 16,23 | 0,00 | 8,77 | 2,11 | 37 | 7 | 10 | 24 | 6 |
| 11,54 | 0,00 | 8,08 | 1,04 | 32 | 7 | 8 | 41 | 6 |
| 13,47 | 0,23 | 4,34 | 1,42 | 36 | 7 | 7 | 29 | 12 |
| 7,62 | 0,00 | 4,29 | 0,00 | 32 | 7 | 7 | 25 | 12 |
| 10,93 | 0,71 | 5,23 | 1,02 | 52 | 7 | 13 | 39 | 17 |
| 12,50 | 0,00 | 9,38 | 0,00 | 32 | 7 | 7 | 20 | 6 |
| 11,33 | 0,36 | 4,68 | 0,81 | 31 | 7 | 7 | 56 | 12 |
| 8,33 | 1,45 | 3,62 | 0,00 | 52 | 8 | 11 | 35 | 12 |
| 10,74 | 0,83 | 6,61 | 0,93 | 43 | 8 | 8 | 51 | 12 |
| 11,26 | 0,54 | 4,83 | 4,62 | 34 | 7 | 8 | 49 | 12 |
| 19,00 | 0,00 | 13,00 | 0,00 | 31 | 8 | 7 | 41 | 12 |
| 10,05 | 0,00 | 8,99 | 1,28 | 32 | 9 | 7 | 53 | 12 |
| 10,29 | 0,98 | 8,82 | 1,24 | 34 | 11 | 7 | 34 | 12 |
| 12,81 | 0,49 | 6,90 | 0,00 | 30 | 7 | 7 | 35 | 12 |
| 11,46 | 0,64 | 10,83 | 0,00 | 44 | 7 | 16 | 30 | 1 |
| 12,12 | 0,00 | 7,41 | 0,00 | 34 | 7 | 9 | 25 | 15 |
| 23,33 | 0,00 | 26,67 | 0,00 | 78 | 14 | 27 | 22 | 12 |
| 13,99 | 0,00 | 11,52 | 5,07 | 62 | 18 | 20 | 24 | 6 |
| 12,09 | 0,00 | 9,89 | 0,00 | 66 | 20 | 13 | 26 | 12 |
| 17,28 | 0,00 | 13,58 | 2,33 | 76 | 17 | 17 | 26 | 12 |
| 16,84 | 0,00 | 10,53 | 2,33 | 62 | 18 | 16 | 28 | 1 |
| 12,90 | 0,00 | 7,53 | 4,02 | 96 | 20 | 29 | 39 | 12 |
| 14,65 | 0,00 | 10,61 | 1,55 | 68 | 13 | 16 | 37 | 6 |
| 21,14 | 0,00 | 9,76 | 4,72 | 58 | 16 | 16 | 57 | 6 |
| 20,00 | 0,00 | 16,00 | 11,11 | 56 | 23 | 9 | 23 | 6 |
| 16,67 | 0,00 | 12,75 | 1,79 | 103 | 23 | 33 | 99 | 1 |
| 12,82 | 0,85 | 5,13 | 0,91 | 44 | 10 | 9 | 33 | 12 |
| 14,06 | 0,26 | 5,99 | 0,59 | 55 | 21 | 8 | 34 | 1 |
| 13,08 | 0,00 | 7,48 | 3,16 | 41 | 10 | 11 | 51 | 12 |
| 11,90 | 0,00 | 5,95 | 0,00 | 71 | 16 | 26 | 43 | 1 |
| 7,81 | 0,00 | 1,56 | 0,00 | 62 | 17 | 20 | 43 | 6 |
| 15,38 | 0,00 | 10,26 | 15,00 | 99 | 20 | 37 | 39 | 1 |
| 18,97 | 0,00 | 13,79 | 5,56 | 90 | 24 | 27 | 32 | 6 |
| 13,11 | 0,00 | 8,25 | 0,93 | 61 | 20 | 16 | 42 | 12 |
| 13,88 | 0,00 | 11,03 | 2,70 | 64 | 23 | 11 | 28 | 6 |
| 12,94 | 0,00 | 4,71 | 3,03 | 68 | 10 | 25 | 34 | 6 |

**S4** **Table 2:**  Regression statistics of the model taking Matrix Sentence (MS) as dependent variable, and PANSS Positive as independent variable, with and without adjusting for Age and/or Education.

| *Regression Statistics without adjusting* | | | | |
| --- | --- | --- | --- | --- |
|  | *Coefficients* | *SE* | *t Stat* | *P-value* |
| Intercept | 9,961 | 1,234 | 8,072 | 0,000 |
| PANSS Positive | 0,273 | 0,088 | 3,092 | 0,004 |
| *Regression Statistics adjusting for Age and Education* | | | | |
|  | *Coefficients* | *SE* | *t Stat* | *P-value* |
| Intercept | 11,265 | 2,378 | 4,736 | 0,000 |
| PANSS Positive | 0,264 | 0,098 | 2,707 | 0,010 |
| Age | -0,021 | 0,038 | -0,570 | 0,572 |
| Education | -0,050 | 0,128 | -0,392 | 0,698 |
| *Regression Statistics adjusting for Age* | | | | |
|  | *Coefficients* | *SE* | *t Stat* | *P-value* |
| Intercept | 10,641 | 1,744 | 6,100 | 0,000 |
| PANSS Positive | 0,279 | 0,090 | 3,109 | 0,004 |
| Age | -0,021 | 0,037 | -0,556 | 0,581 |
| *Regression Statistics adjusting for Education* | | | | |
|  | *Coefficients* | *SE* | *t Stat* | *P-value* |
| Intercept | 10,515 | 1,962 | 5,359 | 0,000 |
| PANSS Positive | 0,259 | 0,096 | 2,690 | 0,011 |
| Education | -0,046 | 0,126 | -0,366 | 0,717 |

**S4** **Table 3:**  Regression statistics of the model taking Matrix Sentence (MS) as dependent variable, and PANSS Total as independent variable, with and without adjusting for Age and/or Education.

| *Regression Statistics without adjusting* | | | | |
| --- | --- | --- | --- | --- |
|  | *Coefficients* | *SE* | *t Stat* | *P-value* |
| Intercept | 9,401 | 1,422 | 6,610 | 0,000 |
| PANSS Total | 0,077 | 0,025 | 3,039 | 0,004 |
| *Regression Statistics adjusting for Age and Education* | | | | |
|  | *Coefficients* | *SE* | *t Stat* | *P-value* |
| Intercept | 11,193 | 2,358 | 4,747 | 0,000 |
| PANSS Total | 0,077 | 0,028 | 2,792 | 0,008 |
| Age | -0,035 | 0,038 | -0,911 | 0,369 |
| Education | -0,067 | 0,124 | -0,539 | 0,593 |
| *Regression Statistics adjusting for Age* | | | | |
|  | *Coefficients* | *SE* | *t Stat* | *P-value* |
| Intercept | 10,371 | 1,780 | 5,826 | 0,000 |
| PANSS Total | 0,082 | 0,026 | 3,160 | 0,003 |
| Age | -0,034 | 0,038 | -0,910 | 0,369 |
| *Regression Statistics adjusting for Education* | | | | |
|  | *Coefficients* | *SE* | *t Stat* | *P-value* |
| Intercept | 10,188 | 2,079 | 4,901 | 0,000 |
| PANSS Total | 0,072 | 0,027 | 2,665 | 0,011 |
| Education | -0,065 | 0,124 | -0,524 | 0,604 |

**S4** **Table 4:**  Regression statistics of the model taking Truncated Non-anomalous Sentence (TS-A) as dependent variable, and PANSS negative as independent variable, with and without adjusting for Age and/or Education.

| *Regression Statistics without adjusting* | | | | |
| --- | --- | --- | --- | --- |
|  | *Coefficients* | *SE* | *t Stat* | *P-value* |
| Intercept | 0,427 | 0,109 | 3,928 | 0,000 |
| PANSS Negative | -0,015 | 0,007 | -2,242 | 0,031 |
| *Regression Statistics adjusting for Age and Education* | | | | |
|  | *Coefficients* | *SE* | *t Stat* | *P-value* |
| Intercept | 0,236 | 0,222 | 1,065 | 0,294 |
| PANSS Negative | -0,013 | 0,008 | -1,712 | 0,095 |
| Age | 0,001 | 0,004 | 0,354 | 0,725 |
| Education | 0,013 | 0,013 | 0,982 | 0,333 |
| *Regression Statistics adjusting for Age* | | | | |
|  | *Coefficients* | *SE* | *t Stat* | *P-value* |
| Intercept | 0,382 | 0,164 | 2,326 | 0,026 |
| PANSS Negative | -0,016 | 0,007 | -2,231 | 0,032 |
| Age | 0,001 | 0,004 | 0,366 | 0,716 |
| *Regression Statistics adjusting for Education* | | | | |
|  | *Coefficients* | *SE* | *t Stat* | *P-value* |
| Intercept | 0,279 | 0,184 | 1,515 | 0,138 |
| PANSS Negative | -0,012 | 0,007 | -1,698 | 0,098 |
| Education | 0,013 | 0,013 | 0,998 | 0,325 |

**S4** **Table 5:**  Regression statistics of the model taking Null Pronoun (Null) as dependent variable, and PANSS Positive as independent variable, with and without adjusting for Age and Education.

| *Regression Statistics without adjusting* | | | | |
| --- | --- | --- | --- | --- |
|  | *Coefficients* | *SE* | *t Stat* | *P-value* |
| Intercept | 5,124 | 1,505 | 3,406 | 0,002 |
| PANSS Positive | 0,283 | 0,107 | 2,628 | 0,012 |
| *Regression Statistics adjusting for Age and Education* | | | | |
|  | *Coefficients* | *SE* | *t Stat* | *P-value* |
| Intercept | 6,927 | 2,865 | 2,418 | 0,021 |
| PANSS Positive | 0,296 | 0,118 | 2,517 | 0,016 |
| Age | -0,053 | 0,045 | -1,163 | 0,252 |
| Education | -0,006 | 0,154 | -0,037 | 0,970 |
| *Regression Statistics adjusting for Age* | | | | |
|  | *Coefficients* | *SE* | *t Stat* | *P-value* |
| Intercept | 6,855 | 2,096 | 3,270 | 0,002 |
| PANSS Positive | 0,298 | 0,108 | 2,764 | 0,009 |
| Age | -0,053 | 0,045 | -1,179 | 0,246 |
| *Regression Statistics adjusting for Education* | | | | |
|  | *Coefficients* | *SE* | *t Stat* | *P-value* |
| Intercept | 5,081 | 2,397 | 2,120 | 0,041 |
| PANSS Positive | 0,284 | 0,118 | 2,409 | 0,021 |
| Education | 0,004 | 0,154 | 0,023 | 0,981 |

**S4** **Table 6:**  Regression statistics of the model taking Null 3Person Referential Pronoun (N3P+R) as dependent variable, and PANSS Positive as independent variable, with and without adjusting for Age and Education and for Education.

| *Regression Statistics without adjusting* | | | | |
| --- | --- | --- | --- | --- |
|  | *Coefficients* | *SE* | *t Stat* | *P-value* |
| Intercept | -1,070 | 1,015 | -1,054 | 0,299 |
| PANSS Positive | 0,245 | 0,072 | 3,379 | 0,002 |
| *Regression Statistics adjusting for Age and Education* | | | | |
|  | *Coefficients* | *SE* | *t Stat* | *P-value* |
| Intercept | 0,080 | 1,954 | 0,041 | 0,967 |
| PANSS Positive | 0,228 | 0,080 | 2,844 | 0,007 |
| Age | -0,010 | 0,031 | -0,328 | 0,745 |
| Education | -0,068 | 0,105 | -0,649 | 0,520 |
| *Regression Statistics adjusting for Age* | | | | |
|  | *Coefficients* | *SE* | *t Stat* | *P-value* |
| Intercept | -0,771 | 1,439 | -0,536 | 0,595 |
| PANSS Positive | 0,248 | 0,074 | 3,350 | 0,002 |
| Age | -0,009 | 0,031 | -0,297 | 0,768 |
| *Regression Statistics adjusting for Education* | | | | |
|  | *Coefficients* | *SE* | *t Stat* | *P-value* |
| Intercept | -0,275 | 1,608 | -0,171 | 0,865 |
| PANSS Positive | 0,226 | 0,079 | 2,860 | 0,007 |
| Education | -0,066 | 0,103 | -0,641 | 0,526 |

**S4** **Table 7:**  Regression statistics of the model taking Null 3Person Referential Pronoun (N3P+R) as dependent variable, and PANSS Total as independent variable, with and without adjusting for Age and Education and for Education.

| *Regression Statistics without adjusting* | | | | |
| --- | --- | --- | --- | --- |
|  | *Coefficients* | *SE* | *t Stat* | *P-value* |
| Intercept | -1,459 | 1,181 | -1,236 | 0,224 |
| PANSS Total | 0,067 | 0,021 | 3,186 | 0,003 |
| *Regression Statistics adjusting for Age and Education* | | | | |
|  | *Coefficients* | *SE* | *t Stat* | *P-value* |
| Intercept | 0,182 | 1,957 | 0,093 | 0,926 |
| PANSS Total | 0,064 | 0,023 | 2,771 | 0,009 |
| Age | -0,021 | 0,032 | -0,655 | 0,517 |
| Education | -0,087 | 0,103 | -0,845 | 0,404 |
| *Regression Statistics adjusting for Age* | | | | |
|  | *Coefficients* | *SE* | *t Stat* | *P-value* |
| Intercept | -0,888 | 1,486 | -0,598 | 0,554 |
| PANSS Total | 0,070 | 0,022 | 3,224 | 0,003 |
| Age | -0,020 | 0,032 | -0,642 | 0,525 |
| *Regression Statistics adjusting for Education* | | | | |
|  | *Coefficients* | *SE* | *t Stat* | *P-value* |
| Intercept | -0,418 | 1,716 | -0,243 | 0,809 |
| PANSS Total | 0,060 | 0,022 | 2,714 | 0,010 |
| Education | -0,086 | 0,102 | -0,839 | 0,407 |
