# Supplementary material for: Grammatical impairment in schizophrenia: An exploratory study of the pronominal and sentential domains
Source: PLoS One. 2023 Sep 12;18(9):e0291446. doi: 10.1371/journal.pone.0291446 (PMC10497169; doi:10.1371/journal.pone.0291446)
Supplement: Attachment — Submitted filename: Response to Reviewers.docx [file pone.0291446.s005.docx]

Michal Ptaszynski, PhD
Academic Editor
PLOS ONE

13 August 2023

Dear Mr. Ptaszynski,

Re: Resubmission of manuscript reference No. PONE-D-23-07587

Firstly, we thank you and both reviewers for the insightful and valuable comments, which contributed to substantial improvements in our research and in the quality of the present manuscript.

Please find attached a revised version of our manuscript entitled *Grammatical impairment in schizophrenia: an exploratory study of the pronominal and sentential domains*, which we resubmit for consideration for publication at PLOS ONE.

In what follows, detailed responses are provided to each of the reviewers’ comments.

Review #1:

1. **“The paper has been meticulously written, although I believe the discussion section could be more concise.”**

Authors: We thank the reviewer for the positive appraisal and for the motivation to be more synthetic. In response to the reviewer’s concern, the discussion section has been reorganized and shortened, particularly regarding our 4 predictions.

2. **“[…] one aspect that this study could further elaborate on is the nature of language and thought disorders in schizophrenia […] It is challenging to ascertain why schizophrenic patients with CBP develop such differences and how these differences correlate with their pathology.”**

Authors: In the revised manuscript, we elaborated on the nature of language and thought disorders in SZ, to highlight the important role of crosslinguistic studies for the characterization of the SZ-specific linguistic profile (see lines 45­­-84; 111-113).

The detailed characterization of the SZ-specific linguistic profile, including how language anomalies manifest crosslinguistically can help us examine (i) whether language dysfunctions be identified in terms of grammatical features, (ii) if it is possible to characterize SZ language symptoms crosslinguistically, and (iii) how such characterization can shed light on human cognition across typical and atypical populations. Moreover, the relevance of crosslinguistic studies in SZ is based on the understanding that, although language neural basis is the same across human populations, languages vary in their repertoire of grammatical choices. In other words, to understand the complexity of language anomalies in SZ and how they relate to pathology, we need to understand how these anomalies are manifested in different languages with different grammatical choices (e.g., wh-fronting, word order and licensing of null pronouns).

Our research was inspired by recent studies adopting linguistic models of SZ that identified grammatical measures of SZ (e.g., syntactic complexity, pronouns, referentiality, definiteness among others.). We examined some of these grammatical measures aiming at providing a more fine-grained analysis, as well as crosslinguistic validation.

We believe that formal characterizations of grammatical deficits are important to our understanding of how SZ affects language production and comprehension. These points were made clearer in the revised manuscript.

3. **“[…] it would be beneficial to provide more detailed information about the characteristics of the patient group. […] and a more comprehensive description of the clinical characteristics of the schizophrenia group (n=20) in Sample 1 would be highly valuable. For instance, could you provide information regarding the dosage of antipsychotic medication?**

Authors: Unfortunately, we did not have access to information concerning the exact dosage of antipsychotic medication taken by our SZ subjects . We are only able to provide information on the duration of the illness (sample 1: years; sample 2: days) and the medication participants were taking when they were interviewed. This limitation was included in the revised manuscript.

All patients with schizophrenia were diagnosed based on standard Structured Clinical Interview for DSM-IV rating SCID (Portuguese version). They also had their symptoms evaluated in terms of the scores of PANSS and BPRS psychometric scales during the clinical interview.

Moreover, as informed in the manuscript, all patients with SZ, on both samples, were medicated and out of the acute psychotic phase at the onset of the study. Of the 20 patients with SZ of the sample 1, 85% (N=17) were under typical antipsychotic medication and 25% (N=5) under atypical antipsychotic medication; whereas of the 11 subjects with schizophrenia of sample 2, 55% (N=6) were under typical antipsychotic medication and 82% (N=9) under atypical antipsychotic medication.

4. **“[…] the correlation analysis, particularly Table 6, it seems necessary to conduct a multiple regression analysis while adjusting for age and educational history.**

Authors: Thank you for this important recommendation, which we duly followed by conducting a multiple linear regression to determine the presence of confounding effect of age and education on the correlations presented on Table 6 of the manuscript.

The presence of confounding effect was determined based on the 10% rule, which determines that more than a 10% difference between the coefficient of X (i.e., the change in the value of dependent variable (Y) corresponding to the unit change in the value of independent variable (X)) of the unadjusted and adjusted models is a signal of confounding effect.

In the revised manuscript we now provide full data of the analyses on S4 Appendix (full regression data), and the results of the regression analyses are shown on items a-f:

a. The model taking Matrix Sentence (MS) as dependent variable, and PANSS Positive as independent variable, when adjusted for Age and/or Education showed 1% change. Thus, the regression analysis showed no confounding effect of Age and/or Education. The overall model of regression taking MS as dependent variable, and PANSS Positive as independent variable was statistically significant (*R^2^*=0,201; *F*(1,38)=9,561; *p*=0,004).
b. The model taking Matrix Sentence (MS) as dependent variable, and PANSS Total as independent variable, when adjusted for Age and Education and for Education only showed 0% change, and when adjusted for Age only showed 1% change. Thus, the regression analysis showed no confounding effect of Age and/or Education. The overall model of regression taking MS as dependent variable, and PANSS Total as independent variable was statistically significant (*R^2^*=0,195; *F*(1,38)=9,237; *p*=0,004).
c. The model taking Truncated Non-anomalous Sentence (TS-A) as dependent variable, and PANSS negative as independent variable, when adjusted for Age and/or Education showed 0% change. Thus, the regression analysis showed no confounding effect of Age and/or Education. The overall model of regression taking TS-A as dependent variable, and PANSS negative as independent variable was statistically significant (*R^2^*=0,117; *F*(1,38)=5,026; *p*=0,031).
d. The model taking Null Pronoun (Null) as dependent variable, and PANSS Positive as independent variable, when adjusted for Age and Education showed 1% change, when adjusted for Age only showed 2% change, and when adjusted for Education only showed 0% change. Thus, the regression analysis showed no confounding effect of Age and/or Education. The overall model of regression taking Null as dependent variable, and PANSS positive as independent variable was statistically significant (*R^2^*=0,154; *F*(1,38)=6,909; *p*=0,012).
e. The model taking Null 3Person Referential Pronoun (N3P+R) as dependent variable, and PANSS Total as independent variable, when adjusted for Age and Education and for Age only showed 0% change, and when adjusted for Education only showed 1% change. Thus, the regression analysis showed no confounding effect of Age and/or Education. The overall model of regression taking N3P+R as dependent variable, and PANSS Total as independent variable was statistically significant (*R^2^*=0,211; *F*(1,38)=10,149; *p*=0,003).
f. The model taking Null 3Person Referential Pronoun (N3P+R) as dependent variable, and PANSS Positive as independent variable, when adjusted for Age and Education and for Education only showed 2% change, and when adjusted for Age only showed 0% change. Thus, the regression analysis showed no confounding effect of Age and/or Education. The overall model of regression taking N3P+R as dependent variable, and PANSS positive as independent variable was statistically significant (*R^2^*=0,231; *F*(1,38)=11,417; *p*=0,002).
5. **“To prevent confusion, it would be less ambiguous to consistently refer to the non-schizophrenic group rather than using "CT" in the text.”**

Authors. Point well-taken. We changed all references to control participants (CT) to participants without SZ (NSZ) in the revised manuscript.

6. **“Considering the depth and significance of the findings, you might want to consider submitting the article to a specialized journal focused on schizophrenia. Doing so could attract even more attention from readers interested in this specific area of research.”**

Authors: That is indeed a possibility. Nevertheless, a journal with a wide range of academic readers might be more suitable to our paper/research given its the interdisciplinary nature. Although a specialized journal would attract specialists in SZ, it would be off the radar of readers interested in linguistics.

Review #2:

1. **“Recent study showed that amisulpride improved language performence compared with placebo in SZ (Zhu et al., Amisulpride augmentation therapy improves cognitive performance and psychopathology in clozapine-resistant treatment-refractory schizophrenia: a 12-week randomized, double-blind, placebo-controlled trial). I suggest the authors should noted it in the background or discussion section.”**

Authors: This is an important observation. Thank you. The revised version of the manuscript (see lines 705-706; and 733-737) provides information on the effect of medication on language dysfunctions in SZ, as well as on the improvement observed in the amisulpride augmentation therapy.

2. **“How to calculate the sample size? Can these sample sizes achieve statistical efficacy?”**

Authors: Thanks for the comment. Accordingly, in the reviewed manuscript (see lines 249-261), we provide information regarding the sample sizes, which were defined in Mota et al. (2014 and 2017) and are based on world and Brazil’s prevalence of schizophrenia.

3. **“Please interpret why the sample is divided into two sample groups. What is the purpose of splitting the sample into two groups in this way? I suggest stating in the paper.”**

Authors: The new version of manuscript (see lines 186-191) provides information on the reason why SZ participants were divided in two groups. It was due to the fact patients in sample 1 had chronic SZ., whereas participants in sample 2 was first episode SZ patients. This split is in line with studies showing structural and functional anomalies in language areas of the brain in patients with chronic SZ, and differences in language production between chronic and first episode SZ (DeLise, 2001; DeLise et al, 2006 among others).

Thank you for your attention, and we look forward to hearing from you.

Sincerely,

Monica Frias Chaves
Department of Letters, Pontifical University of Rio de Janeiro, PUC-Rio

Phone: +55 (21) 3527-1294 /FAX +55 (21) 3527-1296
